# Investigation into the Effect of Thermal Treatment on the Obtaining of Magnetic Phases: Fe_5_Y, Fe_23_B_6_, Y_2_Fe_14_B and αFe within the Amorphous Matrix of Rapidly-Quenched Fe_61+x_Co_10−x_W_1_Y_8_B_20_ Alloys (Where *x* = 0, 1 or 2)

**DOI:** 10.3390/ma13040835

**Published:** 2020-02-12

**Authors:** Petrica Vizureanu, Marcin Nabiałek, Andrei Victor Sandu, Bartłomiej Jeż

**Affiliations:** 1 Faculty of Materials Science and Engineering, GheorgheAsachi Technical University of Iasi, Blvd. D. Mangeron 41, 700050 Iasi, Romania; peviz2002@yahoo.com; 2 Department of Physics, Faculty of Production Engineering and Materials Technology, Częstochowa University of Technology, Al. Armii Krajowej 19, 42-200 Częstochowa, Poland; 3 Romanian Inventors Forum, Str. Sf.P.Movila 3, 700089 Iasi, Romania

**Keywords:** Injection casting method, bulk amorphous alloys, nanocrystalline materials, isothermal annealing, soft magnetic materials

## Abstract

The paper presents the results of research on the structure and magnetic properties of Fe_61+*x*_Co_10−*x*_W_1_Y_8_B_20_ alloys (where *x* = 0, 1 or 2). The alloys were produced using two production methods with similar cooling rates: Injection casting and suction casting. The alloy samples produced were subjected to isothermal annealing at 940 K for 10 min. The structure of the materials was examined using X-ray diffraction. Isothermal annealing has led to the formation of various crystallization products depending on the chemical composition of the alloy and the structure of the alloy in a solidified state. In two cases, the product of crystallization was the hard magnetic phase Y_2_Fe_14_B. However, the mechanism of this phase formation was different in both cases. The magnetic properties of alloys were tested using a vibrating sample magnetometer and a Faraday magnetic balance. It is found that the grain crystallite size of the crystalline phases have a decisive influence on the value of the coercive field (especially in the case of hard magnetic phases). It has been shown that privileged areas can already be created during the production process. Their presence determines the crystallization process.

## 1. Introduction

Modern materials are expected to fulfil increasingly demanding requirements to warrant their adoption in applications. Currently, the main requirements are efficiency and the protection of the natural environment. Many modern materials are being used in very specialist fields due to their desirable properties. Iron-based materials are used, amongst others, in the power and electrochemical industries. These materials are mostly magnetic alloys which feature soft magnetic properties. Materials exhibiting soft magnetic properties are characterized by a low-value coercive field, low core losses and high saturation magnetization. Such properties are exhibited, among others, by amorphous [1,2,3,4,5,6] and nanocrystalline iron-based alloys [7,8,9,10]. These parameters are especially important for such applications as magnetic cores, where efficient demagnetization is required. The magnetic properties of the material depend not only on its chemical composition, but also on the production process, and the purity of the component elements. One production method, suitable for obtaining materials with soft magnetic properties, is the “rapid quenching” of molten iron-based compounds. The rapid solidification of the liquid alloy “freezes” the disordered atomic structure, which is characterized by the absence of a long-distance atomic order [11,12].

The first solid amorphous alloys in the form of ribbons were produced in the 1960s [13,14]. In the beginning, the production methods only allowed the manufacture of amorphous alloys in the form of very thin tapes of an approximate thickness of 30 μm, which substantially restricted their applicability. For more than 20 years, research work tried to establish production processes and conditions for obtaining amorphous alloys of thicknesses greater than 100 μm [15]. Finally, A. Inoue [7] formulated three criteria which allowed the production of amorphous material samples with greater thicknesses and using a lower quenching speed than that used for the production of the classical amorphous ribbons [16,17,18,19]. Currently, there are several production methods which allow the production of amorphous materials; two examples are the “injection-casting” and “suction-casting” methods. In these methods, the liquid alloy is injected (or drawn, in the suction-casting method) into a water-cooled copper die. The quenching speed used in these methods reaches 10^1^–10^3^ K/s [20,21,22,23]. Given that a suitable chemical composition is used, these methods allow the production of a disordered structure. Material with a partially crystallized structure (and a size of crystalline grains of up to 100 nm)—i.e., so-called “nanocrystalline” material—is difficult to obtain during a single-step production process. However, under certain conditions, it is possible to obtain a nanocrystalline alloy in the course of the rapid solidification method [24]. It is difficult to describe precisely the production process, due to its low repeatability. Therefore, the technique has to be adapted for each chemical composition. The nanocrystallization of an alloy could also be achieved using an appropriate thermal treatment of the amorphous alloy [25,26]. Depending on the time duration and temperature of the annealing process, the structure of an amorphous alloy will either become relaxed, or it will change into a two-phase structure consisting of an amorphous matrix with crystalline grains. In order to obtain crystalline grains, the annealing process has to be performed closeto the crystallization temperature of the alloy. The duration of the annealing process affects the size and number of the created crystalline grains. The nanocrystalline alloys, resulting from appropriate thermal treatment, are characterized by good soft magnetic properties [4,27]. The amorphous materials with soft magnetic properties are based on iron, cobalt, or nickel. In addition to the transition metals, whose contribution comprises more than 50% of the alloy, selected non-metallic elements are used; e.g., boron, silicon or germanium. The selection of elements for the chemical composition of an alloy is not haphazard. A high iron and/or cobalt content ensures good magnetic properties. The addition of boron in an appropriate proportion to iron ensures a good glass-forming ability (GFA) of the alloy. The addition of yttrium is also known to improve GFA, as well as yielding an improvement in the magnetic properties [28]. The addition of tungsten has a positive effect on the soft magnetic properties of the alloy [29]. According to the Turnbull theorem [30], this chemical composition of the alloy fulfils the relation T_rg_ = T_g_/T_l_, (T_rg_: Reduced glass transition temperature; T_g_: Glass transition temperature; T_l_: Liquid temperature) where T_rg_ is approximately 0.6, and the alloy has a eutectic composition.

To summarize, the aim of this work was to investigate the structure and magnetic properties of rapidly-quenched Fe_61+x_Co_10−x_W_1_Y_8_B_20_alloys (where x = 0, 1 or 2) in the amorphous, as-quenched state, as well as the state following thermal treatment. Above all, we wanted to check how a small change in the chemical composition of the alloy produced by different methods affects the creation of crystalline phases in the annealing process.

## 2. Materials and Methods

The component elements for the alloys were weighed to an accuracy of up to 0.0001 g. Each of the samples weighed 10 g. The weighed components were melted in an arc-furnace (Author’s device, Częstochowa, Poland), under a protective argon atmosphere. The samples were placed in the working chamber—a cavity within a copper die—and were melted using a plasma arc. The temperature of the arc was controlled by the current passing though the electrode. Before this operation, the re-melting of a titanium getter was undertaken, in order to remove any remaining oxygen and other impurities from the working chamber. The first melting of the material was undertaken using a lower electrode current to avoid material splatter. Each ingot was re-melted several times, in order to achieve a better homogeneity of the resulting alloy. After each melting process, the ingot was inverted by means of a manipulator, and this action further aided the mixing of the component elements. After this process, each ingot was divided into smaller fragments, and subjected to mechanical and ultrasonic bath cleaning.

The alloys used in this study were made by injection and suction-casting methods, in conjunction with a water-cooled copper die. These methods allowed the achievement of rapid cooling speeds of 10^1^–10^3^ K/s. The casting process was performed under a stable argon pressure and using identical copper dies. The samples were cast in the form of rectangular plates with the following approximate dimensions: Width = 5 mm, length = 10 mm, and thickness = 0.5 mm. Five plate samples were made for each alloy composition.

Separate X-ray diffraction (Bruker, Billerica, MA, USA) studies were performed for each of the tested plates. Tests were carried out on the surface of the samples and after crushing. Then, powders were mixed for each of the examined alloys and X-ray examinations were performed. The obtained results coincided with high accuracy with the diffraction obtained for the samples from individual plates.

**Figure 1:**
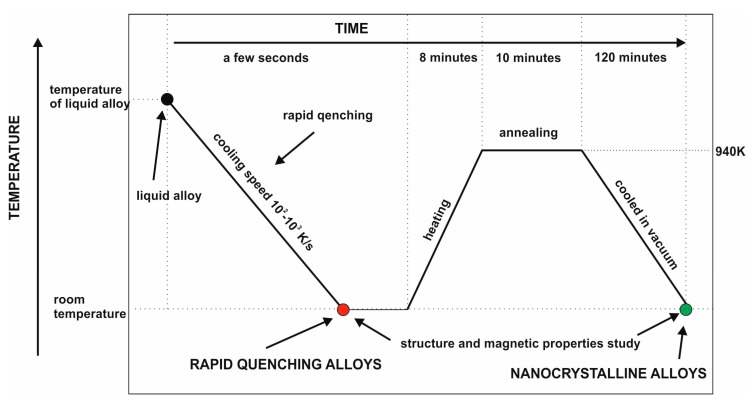
Thermal diagram of tested alloys.

Following casting, the resulting samples were crushed using a low-energy process. The powdered samples were subjected to an isothermal annealing process in a vacuum at a temperature of 940 K for 10 min. The process of the thermal treatment of samples consisted of three stages: heating (8 min), annealing (10 min) and cooling (120 min). The samples enclosed in a quartz tube were placed in an oven (author’s device, Częstochowa, Poland) preheated to 940 K. After 8 min, the sample reached oven temperature. According to the established parameters, the sample was annealed for 10 min. After this time, the oven chamber was opened and the sample was cooled to room temperature (Figure 1). The heat treatment process designed in this way affects the relaxation processes which occur in the sample volume.

The effect of the low-energy crushing on the possible segregation of alloying elements was determined using EDX analysis. A microscope was used, which was the Supra 25 Zeiss Detector SE (Oberkochen, Germany). The structure of the investigated alloys was studied by performing X-ray diffractometry on the powdered samples. A Bruker Advance D8 X-ray diffractometer (Bruker, Billerica, MA, USA) equipped with a CuKα radiation source, was used for this part of the investigation. The magnetic properties were studied by means of a LakeShore vibrating sample magnetometer (Carson, CA, USA) using an external magnetic field of up to 1.7 T. Based on the recorded static hysteresis loops, the values of the coercivity and magnetization of the samples were determined. The thermal stability of the alloys was studied using a Faraday balance (AGH, Kraków, Poland).

## 3. Results

**Figure 2:**
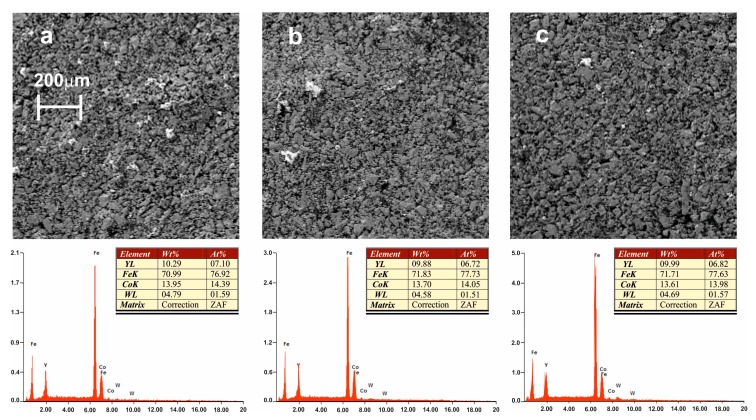
Structure and chemical composition of alloys produced by the suction method: (**a**) Fe_61_Co_10_W_1_Y_8_B_20_, (**b**) Fe_62_Co_9_W_1_Y_8_B_20_, (**c**) Fe_63_Co_8_W_1_Y_8_B_20_.

Figure 2 presents SEM images for samples in the form of powder along with EDX analysis. EDX analysis was carried out for areas with a size of 10 μm. Crushing did not change the chemical composition and separation of alloy components.

**Figure 3:**
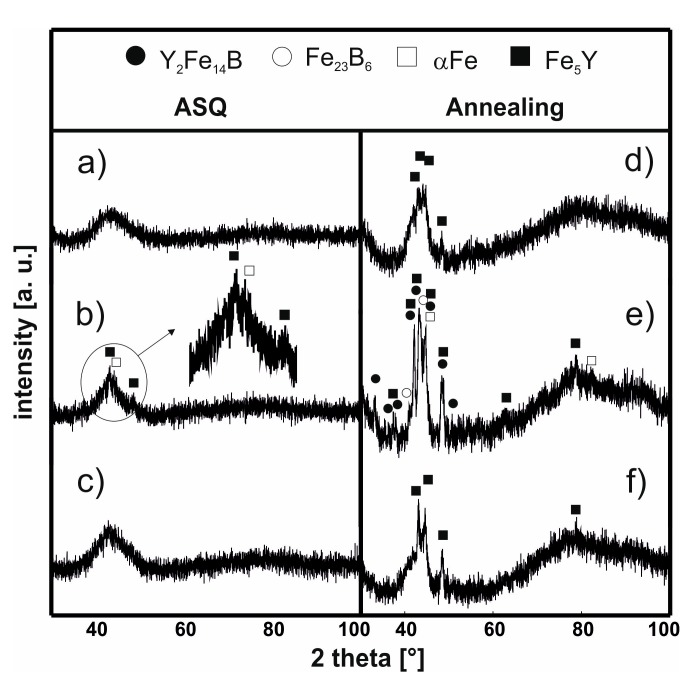
X-ray diffraction patterns for the alloys in the as-cast state (**a**–**c**) and following the thermal treatment at 940 K for 10 min (**d**–**f**) produced using the injection-casting method: Fe_61_Co_10_W_1_Y_8_B_20_, (**a**,**d**), Fe_62_Co_9_W_1_Y_8_B_20_, (**b**,**e**) Fe_63_Co_8_W_1_Y_8_B_20_ (**c,f**).

**Figure 4:**
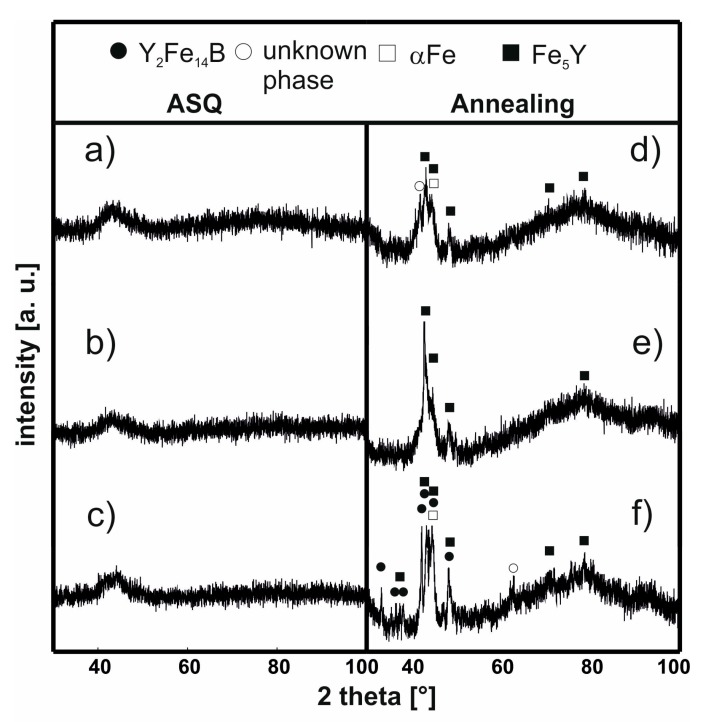
X-ray diffraction patterns for the alloys in the as-cast state (**a**–**c**) and following the thermal treatment at 940 K for 10 min (**d**–**f**) produced using the suction-casting method: Fe_61_Co_10_W_1_Y_8_B_20_, (**a,d**), Fe_62_Co_9_W_1_Y_8_B_20_,(**b,e**) Fe_63_Co_8_W_1_Y_8_B_20_ (**c,f**).

In Figure 3 and Figure 4, X-ray diffraction patterns are presented for the samples of the investigated alloys using injection and suction casting in the as-cast state and following the thermal treatment (940 K/10 min).

The production method resulted in an amorphous structure in two samples, as confirmed by the resulting X-ray patterns (Figure 3a,c). However, the sample of the Fe_62_Co_9_W_1_Y_8_B_20_ alloy (Figure 3b), which was made under the same conditions, shows the presence of a small number of grains of the Fe_5_Y and αFe crystalline phases. After the isothermal annealing process, conducted at a temperature close to the crystallization temperature [31] of the alloy for 10 min, the X-ray diffraction patterns for all of the samples revealed sharp, relatively narrow, peaks, indicating the presence of crystalline phases. In the sample of the Fe_61_Co_10_W_1_Y_8_B_20_ alloy (Figure 3d) and the Fe_63_Co_8_W_1_Y_8_B_20_ alloy (Figure 3f), the presence of two crystalline phases was detected: αFe and Fe_5_Y. This suggests that the crystallization process occurred at similar activation temperatures for these two samples. In the case of the sample of Fe_62_Co_9_W_1_Y_8_B_20_alloy (Figure 3b), which was made by the injection-casting method and partially crystallized during the production process, four crystalline phases can be seen after annealing (Figure 3e). In contrast with the other two samples, in this alloy, after the annealing process, grains of the crystalline phases Y_2_Fe_14_B and Fe_23_B_6_ were observed. The first crystalline phase has been described in the literature as a magnetically hard phase [32], while the second phase is a meta-stable crystalline phase, which is often found during the initial stage of crystallization in the amorphous Fe–Co–B alloys.

For all the investigated alloy samples made using the suction-casting method and still in the as-cast state, the X-ray diffraction patterns revealed only a single broad maximum in close proximity to the 45° 2θ angle; independently of the angular position, the background could be observed. After these samples had been subjected to the thermal treatment at 940 K for 10 min, high-intensity X-ray diffraction rings originating from four different crystalline phases could be seen. For the Fe_61_Co_10_W_1_Y_8_B_20_alloy (Figure 4d) and the Fe_62_Co_9_W_1_Y_8_B_20_ alloy (Figure 4e), within the volume of each sample, crystalline grains of three crystalline phases could be found: Fe_5_Y, αFe and one phase which could not be identified. For the third sample of Fe_63_Co_8_W_1_Y_8_B_20_, (Figure 4f), the annealing process led to the creation of an additional magnetically hard crystalline phase, namely Y_2_Fe_14_B. Based on the Scherrer equation, the average sizes of the crystalline phase grains were determined (please see Table 1).

In the case of the Fe_62_Co_9_W_1_Y_8_B_20_ alloy in the as-cast state, the application of the Scherrer equation is more difficult, as this method should be used to estimate the size of crystallite with a diameter much larger than a few nanometers. For the isothermally annealed samples made by both methods, the grain crystallite size of the Fe_5_Y phase is similar. This means that, in this case, the crystallite size is independent of the production method. In the case of the αFe phase, there is a relationship between the iron content and the crystallite size: with increasing iron content in the alloy, there is an increase in the average crystallite size of the αFe phase. In addition, it could be stated that, in this case, the production method itself influences the crystallite size. A smaller average size of the αFe crystalline phase was observed for the sample made by the suction-casting method. The creation of the magnetically hard Y_2_Fe_14_B and soft Fe_23_B_6_ crystalline phases is very interesting. There is a difference in the creation of the Y_2_Fe_14_B phase between the samples made by the injection and suction-casting methods. It turns out that, despite slight changes in the chemical composition and a similar cooling rate in the volume of alloys, there is a certain segregation of atoms. This arrangement is difficult to predict. The creation of this phase is related to the method of placing the liquid alloy in a copper form. This may result in the formation of various crystalline phases in the annealing process despite the identical composition of the alloy. For the as-cast sample of Fe_62_Co_9_W_1_Y_8_B_20_ made by injection-casting, the size of the Fe_5_Y andαFe crystalline grains was found to be very small. As suggested by many Mössbauer studies of FeCoB [26,33] alloys, there are areas of differing iron concentrations present within the volume of these materials; that is, within the amorphous matrix, some areas are more favorable for the creation of crystalline grains. It can be assumed from the iron-rich amorphous matrix (the low-field component from the Mössbauer spectra) that iron and yttrium were precipitated to create the Fe_5_Y and αFe phases. The re-grouping of the iron and yttrium atoms within the alloy volume in certain preferred regions contributed towards the formation of areas where the atomic arrangement favors the creation of the different phases (which could be the high-field component in the Mössbauer spectra [26,34]).

As a result of the annealing process, atoms diffuse in the volume of alloys. This creates areas with lower internal energy: The nuclei of crystalline phase grains.

The migration of large atoms of yttrium within the amorphous alloy causes the collective movement of different, smaller atoms; in particular, boron.

A good example illustrating the impact of the production method on the structure and magnetic properties of amorphous alloys is its effect on the Curie temperature. In amorphous alloys produced using the injection-casting method, Tc is usually a few degrees Kelvin lower than in the same alloys produced by the suction-casting method [35,36]. Despite the fact that both methods give the possibility of producing amorphous material, these materials have a different magnetic structure. This means that the way of introducing the sample into the copper mold affects the distribution of magnetic atoms in volume. This reasoning confirms that, in the volume of amorphous samples, various clusters can be formed, which are conducive to forming various types of crystalline phases.

**Figure 5:**
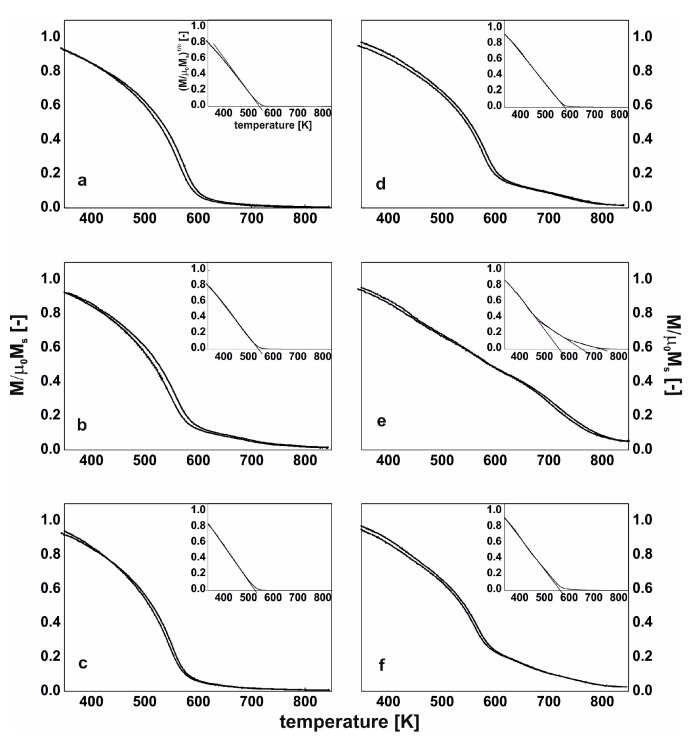
The magnetic saturation polarization curves for the alloys in the as-cast state (**a**–**c**) and after the thermal treatment at 940 K for 10 min (**d**–**f**) made by the injection-casting method: Fe_61_Co_10_W_1_Y_8_B_20_ (**a**,**d**), Fe_62_Co_9_W_1_Y_8_B_20_ (**b**,**e**), Fe_63_Co_8_W_1_Y_8_B_20_ (**c**,**f**).

**Figure 6:**
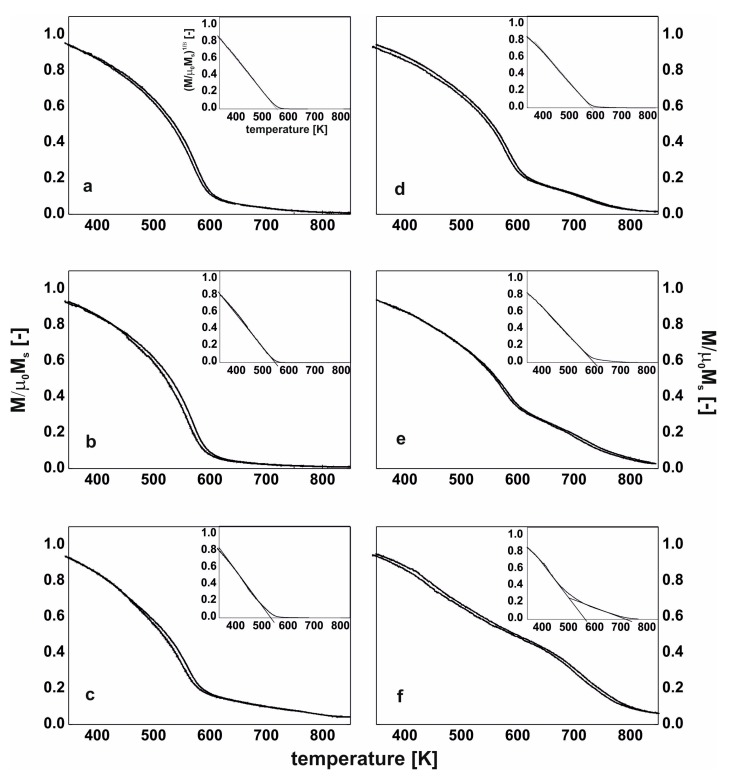
The magnetic saturation polarization curves for the alloys in the as-cast state (**a**–**c**) and after the thermal treatment at 940 K for 10 min (**d**–**f**) made by the suction-casting method: Fe_61_Co_10_W_1_Y_8_B_20_ (**a**,**d**), Fe_62_Co_9_W_1_Y_8_B_20_,(**b**,**e**), Fe_63_Co_8_W_1_Y_8_B_20_ (**c**,**f**).

Therefore, it is possible that, as a result of the thermal treatment of the Fe_62_Co_9_W_1_Y_8_B_20_ alloy, the aforementioned migration occurs, and the Y_2_Fe_14_B and Fe_23_B_6_ phases are created. The Fe_23_B_6_ phase is the so-called pre-natal, metastable phase, in which nuclei or clusters may be created during the rapid solidification process [36]. However, due to their small sizes, they cannot always be identified during X-ray diffractometry studies. In the case of the Fe_63_Co_8_W_1_Y_8_B_20_ alloy produced by the suction-casting method, the mechanism for the creation of this phase is similar. However, it has to be highlighted that the alloy in the as-cast state has an amorphous structure, and therefore there is lack of preferred areas. In this case, the rapid solidification process has not resulted in the nucleation or creation of clusters of the Fe_23_B_6_ phase. The thermal stability of the magnetic saturation polarization is a very important parameter for describing soft magnetic properties. From the analysis of the μ_0_M_s_–T curves (measured at a constant magnetic field of 0.7 T), it could be seen that the individual phases which are present in the volume of the sample affect its thermal stability. In Figure 5 and Figure 6, the thermomagnetic curves, measured over the temperature range from room temperature up to 850 K, are presented. The heating speed was 10 K/min and curves were recorded in both directions. The shapes of the magnetic saturation polarization curves for all of the samples in the as-cast state are similar. However, for the samples of the injection-cast Fe_62_Co_9_W_1_Y_8_B_20_ alloy and the suction-cast Fe_63_Co_8_W_1_Y_8_B_20_ alloy, the area under the curve (with a bend on the curve at around 580 K) is significantly higher than for all the other investigated samples. These small changes in the shape of the thermomagnetic curves are related to the setup of the amorphous structure. As has been mentioned previously, the conglomerates become the areas which favor the creation of crystalline phases.

Due to the fact that amorphous alloys are characterized by a chaotic ordering of atoms, volume areas with different concentrations of atoms can be formed. Such a state may even be associated with the formation of several different magnetic phases in the amorphous structure.

A higher value of saturation magnetic polarization for the injection-cast Fe_62_Co_9_W_1_Y_8_B_20_ alloy and the suction-cast Fe_63_Co_8_W_1_Y_8_B_20_ alloy can be explained by the presence of additional magnetic ordering in the volume of these alloys. This ordering is a contribution to the creation phase Y_2_Fe_14_B after the annealing process.

In the studied temperature range, as a result of the supply of thermal energy, atomic diffusion occurs within the system, which is evident as a difference in the shape of the μ_0_M–T curves, taken in the directions of the heating up and cooling down of the sample. There is a major difference in the shape of the thermomagnetic curves for the samples subjected to thermal treatment. The crystallization products—in the form of crystalline grains—affect the shape of the curve. It was noticed that, in the samples of the injection-cast Fe_62_Co_9_W_1_Y_8_B_20_ alloy and the suction-cast Fe_63_Co_8_W_1_Y_8_B_20_ alloy, the contribution of the hard-magnetic phase increased at the expense of the amorphous matrix. On the assumption that the investigated magnetic samples fulfil Heisenberg assumptions, and with a critical coefficient of β = 0.36, the Curie temperatures for the investigated alloys were calculated (limited to 850 K by the equipment capability). Inserts show the linear fits of the μ_0_M^(1/β)^ curves and determined T_C_ (inserts to Figure 5 and Figure 6).

In the alloys in which the hard-magnetic phase was absent, over the studied temperature range, only the Curie temperature of the amorphous matrix was determined. In the cases of the further discussed alloys (Fe_62_Co_9_W_1_Y_8_B_20_ and Fe_63_Co_8_W_1_Y_8_B_20_), the Curie temperature of the Y_2_Fe_14_B phase was also determined. It has to be noted that, in the case of the amorphous materials, it is only possible to talk about the average value of the Curie temperature, as the process occurs within a narrow temperature range. In the case of the as-cast samples, it is evident that the production method itself influences the value of T_C_ (Table 2). For the injection-cast samples, the T_C_ was found to be a few degrees Kelvin lower than for the suction-cast samples [36,37]. The chemical composition of the alloy also affected the value of T_C_. With an increase in Co content, the Curie temperature of the alloy was slightly higher. For the Fe_62_Co_9_W_1_Y_8_B_20_ alloy, the T_C_ values related to the Y_2_Fe_14_B and Fe_23_B_6_ crystalline phases were determined: they were found to be 580 K, and 680 K, respectively. For the suction-cast Fe_63_Co_8_W_1_Y_8_B_20_ alloy, the T_C_ corresponding to the phase was found to be 595 K. Analyzing further the μ_0_M^(1/β)^ curves for the samples of the Fe_62_Co_9_W_1_Y_8_B_20_ and Fe_63_Co_8_W_1_Y_8_B_20_ alloys, the T_C_ for the bend on the curve above 700 K has to be considered. Neither of the aforementioned crystalline phases has a T_C_ of this value. The degree of crystallization of these samples is quite substantial (Figure 3e and Figure 4f), and neither of the identified crystalline phases contain Co. This could suggest that the described T_C_ in the vicinity of 700 K is the Curie temperature of the remaining amorphous matrix, which is rich in Co.

**Figure 7:**
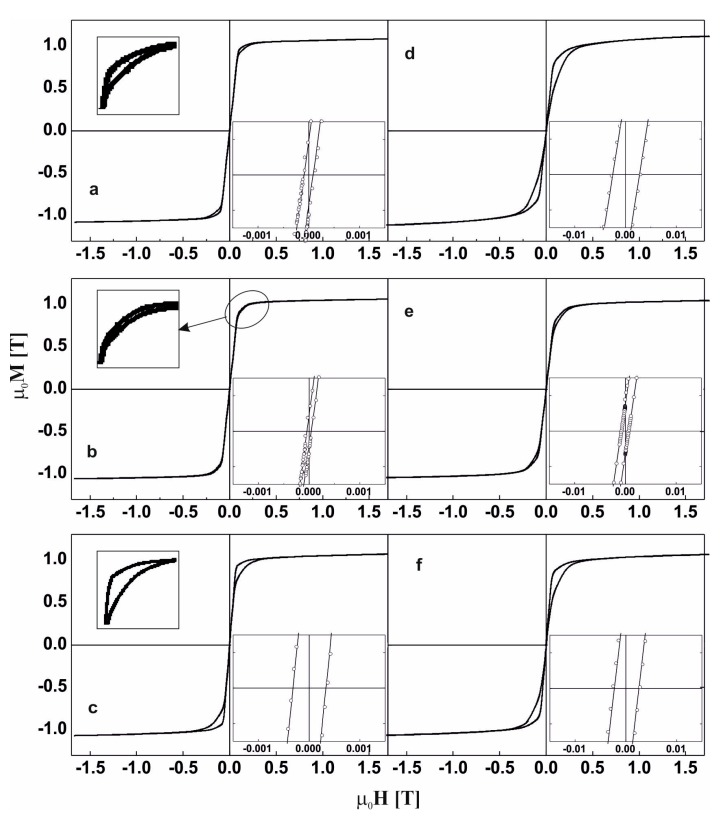
The static magnetic hysteresis loops for the alloys in the as-cast state (**a**–**c**) and after the thermal treatment at 940 K for 10 min (**d**–**f**) made by the injection-casting method: Fe_61_Co_10_W_1_Y_8_B_20_, (**a**,**d**), Fe_62_Co_9_W_1_Y_8_B_20_, (**b**,**e**) Fe_63_Co_8_W_1_Y_8_B_20_ (**c**,**f**).

**Figure 8:**
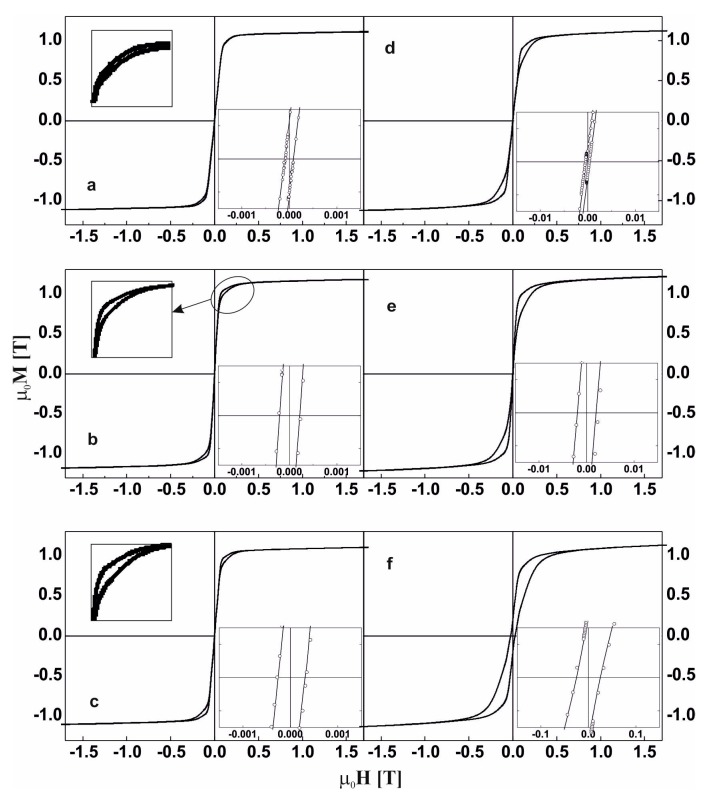
The static magnetic hysteresis loops for the alloys in the as-cast state (**a**–**c**) and after the thermal treatment at 940 K for 10 min (**d**–**f**) made by the suction-casting method: Fe_61_Co_10_W_1_Y_8_B_20_, (**a**,**d**), Fe_62_Co_9_W_1_Y_8_B_20_,(**b**,**e**), Fe_63_Co_8_W_1_Y_8_B_20_ (**c**,**f**).

Figure 7 and Figure 8 reveal the static hysteresis loops for the studied samples. Based on the analysis of the static hysteresis loops, saturation magnetic polarization and coercivity values were determined, and these are given in Table 3. After further analysis, the differences in the magnetization curves depending on the “direction” (magnetizing/demagnetizing) of the external magnetic field were highlighted, and these are shown in the respective inserts in Figure 7a–c and Figure 8a–c.

In the literature, this shape of hysteresis loop is called the “wasp” shape [38,39]. In the initial stages of growth of the magnetically hard phases, the widening of the hysteresis loop at the beginning of the M-H system was not observed. However, the presence of the phases affected the width of the loop at higher values of the external magnetic field.

For the samples in the as-cast state, there is generally a visible increase in the value of the coercive field with decreasing Co content in the alloy; the injection-cast Fe_62_Co_9_W_1_Y_8_B_20_ alloy is an exception. The decrease in the value of the H_C_ is related to the presence of small crystalline grains of the Fe_5_Y and αFe phases (Figure 3b).

In soft magnetic materials, the crystallite size is decisive in the case of the saturation magnetic polarization or coercive field [40]. The presence of small grains does not always lead to improved soft magnetic properties in nanocrystalline ferromagnetics. The results of the study published in [41] prove that the presence of a hard (or semi-hard magnetic phase) in relation to the soft magnetic phase can reduce the coercive field value.

The annealing process caused a major increase in the value of coercive field. Such a high coercive field value compared to the other samples tested is associated with the presence of Y_2_Fe_14_B hard magnetic phase grains in the alloy volume. In the sample of Fe_62_Co_9_Y_8_W_1_B_20_ alloy produced by the injection method and subjected to the annealing process, the crystallites of this phase were characterized by a smaller average dimension. In addition, the presence of other crystalline phases was identified in this sample, including two with soft magnetic properties, which resulted in a lower total coercive field value despite the presence of a hard magnetic phase.

## 4. Conclusions

In this work, the results of an investigation into the structure and magnetic properties of rapidly-quenched iron-based alloys are presented. Samples of the alloys Fe_61+*x*_Co_10−*x*_W_1_Y_8_B_20_ (where *x* = 0, 1 or 2) were made using two different production methods. The obtained materials were subjected to thermal treatment at a temperature of 940 K for 10 min. Each of the investigated alloys was made several times, and the results of the studies have a high repeatability.

Based on previous work by the authors concerning alloys with similar chemical compositions [42], it was found that their crystallization process is primary. This means that, after the crystallization process, there are at least two different crystallization products—the amorphous phase and the crystalline phase—with different chemical compositions.

Based on the aforementioned results, there are certain conclusions which can be drawn:

- The injection and suction-casting methods (into a copper die) allow the manufacture of bulk amorphous alloys with the chemical composition Fe_61+*x*_Co_10−*x*_W_1_Y_8_B_20_ (x = 0, 2).
- In the case of the alloy Fe_62_Co_9_W_1_Y_8_B_20_ in the as-cast state, the presence of a small amount of the phase Fe_5_Y was observed; this could be related to the minor difference in the process time between the two production methods: for the injection-casting method, the time is marginally longer, which might lead to the formation of crystallites during the production process.
- The isothermal annealing process, carried out at a temperature close to the crystallization temperature (940 K/10 min), led to the partial crystallization of the alloys.
- Based on the obtained magnetic test results—i.e., the thermomagnetic curves and static hysteresis loops—it was found that the tested alloys were magnetically heterogeneous materials.
- The Curie temperature values were found to be slightly higher for the suction-cast alloys, which could be related to the different degrees of disorder in the structure.
- The magnetic properties of the investigated alloys in the as-cast state were found to be similar, regardless of the chosen production method.
- The low value of coercivity for the injection-cast Fe_62_Co_9_W_1_Y_8_B_20_ alloy is related to the presence of grains of the Fe_5_Y crystalline phase.
- The carefully designed parameters of the production process for the rapidly-quenched alloys have an influence on the obtaining of fine grains of crystalline phases within the amorphous matrix during the solidification process.
- An increase in the Fe content, at the expense of the Co content, resulted in a decrease in the Curie temperature.
- The size of the crystalline grains was found to exert a strong influence on the value of the coercive field in terms of an increase in the crystallite size hindering the magnetization process, which was visible from the high coercivity value for the suction-cast Fe_63_Co_8_W_1_Y_8_B_20_ alloy. Despite the presence of the Y_2_Fe_14_B phase in the volume of the injection-cast Fe_62_Co_9_W_1_Y_8_B_20_ alloy, a low coercivity was achieved after annealing. This is connected with the relatively small crystallitesize of this phase and the presence of the Fe_23_B_6_ soft magnetic phase.

## Figures and Tables

**Table 1:** Estimated average size of the crystalline grains for the alloys subjected to the thermal treatment (estimated error ~2 nm).

Method	Alloy	Average Crystallite Size (nm)			
		Fe_5_Y	αFe	Y_2_Fe_14_B	Fe_23_B_6_
Injection	Fe_61_Co_10_Y_8_W_1_B_20_	15.0	19.0	-	-
	Fe_62_Co_9_Y_8_W_1_B_20_	24.2	22.5	17.0	14.0
	ASQ Fe_62_Co_9_Y_8_W_1_B_20_	8	8	-	-
	Fe_63_Co_8_Y_8_W_1_B_20_	27.3	26.5	-	-
Suction	Fe_61_Co_10_Y_8_W_1_B_20_	12.0	15.0	-	-
	Fe_62_Co_9_Y_8_W_1_B_20_	26.7	15.4	-	-
	Fe_63_Co_8_Y_8_W_1_B_20_	26.8	25.6	24.8	-

**Table 2:** The Curie temperatures for the injection and suction-cast alloys Fe_61+x_Co_10–x_W_1_Y_8_B_20_ (where *x* = 0, 1 or 2) in the as-cast state (estimated error ~3 K).

Method	Injection Casting				Suction Casting		
	Curie Temperature [K]						
Alloy	ASQ [35]	940 K			ASQ [35]	940 K	
		Y_2_Fe_14_B	Fe_23_B_6_	amorp.		Y_2_Fe_14_B	amorp.
Fe_61_Co_10_Y_8_W_1_B_20_	561	-	-	595	568	-	597
Fe_62_Co_9_Y_8_W_1_B_20_	549	580	655	726	557	-	600
Fe_63_Co_8_Y_8_W_1_B_20_	541	-	-	570	545	595	710

**Table 3:** The values of saturation magnetic polarization and the coercive field for the investigated alloys.

Method	Alloy	Saturation Magnetic Polarization (T)		Saturation Magnetic Polarization for 0.7 T (T)		Coercive Field (A/m)	
		ASQ [35]	940 K	ASQ [35]	940 K	ASQ [35]	940 K
Injection-casting	Fe_61_Co_10_Y_8_W_1_B_20_	1.11	1.11	1.10	1.09	71	2148
	Fe_62_Co_9_Y_8_W_1_B_20_	1.09	1.04	1.08	1.04	33	495
	Fe_63_Co_8_Y_8_W_1_B_20_	1.10	1.06	1.09	1.04	241	1987
Suction-casting	Fe_61_Co_10_Y_8_W_1_B_20_	1.14	1.12	1.13	1.12	61	293
	Fe_62_Co_9_Y_8_W_1_B_20_	1.21	1.22	1.20	1.20	159	1565
	Fe_63_Co_8_Y_8_W_1_B_20_	1.13	1.13	1.12	1.11	223	19820

## References

[1] Geng Y., Zhang Z., Wang Z., Wang Y., Qiang J., Dong C., Wang H., Tegus O. (2016). Magnetic properties and a structure model for high Fe-content Fe–B–Si–Zr bulk glassy alloys. J. Non Cryst. Solids.

[2] Jaafari Z., Seifoddini A., Hasani S. (2019). Enhanced Mechanical and Magnetic Properties of [(Fe_0.9_Ni_0.1_)_77_Mo_5_P_9_C_7.5_B_1.5_]_99.9_Cu_0.1_ Bulk Metallic Glass by Partial Annealing. Metall. Mater. Trans. A.

[3] Han Y., Ding J., Kong F.L., Inoue A., Zhu S.L., Wang Z., Shalaan E., Al-Marzouki F. (2017). FeCo-based soft magnetic alloys with high Bs approaching 1.75 T and good bending ductility. J. Alloy Compd..

[4] Han Y., Inoue A., Kong F.L., Chang C.T., Shu S.L., Shalaan E., Al-Marzouki F. (2016). Softening and good ductility for nanocrystal-dispersed amorphous FeCoB alloys with high saturation magnetization above 1.7 T. J. Alloy Compd..

[5] Wang J., Liu Z.W., Zheng Z.G., Yu H.Y., Tang G.P., Zeng D.C. (2015). Effect of rare earth additions on microstructure, thermal stability and crystallization behavior of melt spun Fe_80.65_Cu_1.35_Si_2_B_14_RE_2_ (RE = Y, Gd, Tb and Dy) soft magnetic alloys. Mater. Lett..

[6] Ackland K., Masood A., Kulkarni S., Stamenov P. (2018). Ultrasoftmagnetic Co-Fe-B-Si-Nb amorphous alloys for high frequency power applications. AIP Adv..

[7] Inoue A. (2000). Stabilization of metallic supercooled liquid and bulk amorphous alloys. Acta Mater..

[8] McHenry M.E., Willard M.A., Laughlin D.E. (1999). Amorphous and nanocrystalline materials for applications as soft magnets. Prog. Mater. Sci..

[9] Li X., Wang Z., Duan H. (2019). The effect of the minor Al addition on microstructure and soft magnetic properties for (Fe_0.5_Co_0.5_)_73.5_Si1_3.5_Nb_3_Cu_1_B_9_ nanocrystalline alloy. J. NonCryst. Solids.

[10] Nabialek M., Jez B., Jez K. (2018). The influence of the method of producing massive amorphous materials on the defects of the structure in the alloy Fe_61+x_Co_10-x_Y_8_W_1_B_20_ where x = 0 or 1. Rev. Chim..

[11] Błoch K., Nabiałek M. (2015). Approach to Ferromagnetic Saturation for the Bulk Amorphous Alloy: (Fe_0:61_Co_0:10_Zr_0:025_Hf_0:025_Ti_0:02_W_0:02_B_0:20_)_97_Y_3_. Acta Phys. Pol. A.

[12] Klement W., Willens R.H., Duwez P. (1960). Non-crystalline structure in solidified Gold-Silicon alloys. Nature.

[13] Duwez P., Willens R.H., Crewdson R.C. (1965). Amorphous phase in palladium—Silicon alloys. J. Appl. Phys..

[14] Chen H.S. (1974). Thermodynamic considerations on the formation and stability of metallic glasses. Acta Metall..

[15] Wang A., Zhao C., Men H., He A., Chang C., Wang X., Li R. (2015). Fe-based amorphous alloys for wide ribbon production with high Bs and outstanding amorphous forming ability. J. Alloy Compd..

[16] Han Y., Chang C.T., Zhu S.L., Inoue A., Louzguine-Luzgin D.V., Shalaan E., Al-Marzouki F. (2014). Fe-based soft magnetic amorphous alloys with high saturation magnetization above 1.5 T and high corrosion resistance. Intermetallics.

[17] Li J., Liu X., Zhao S., Ding H., Yao K. (2015). Fe-based bulk amorphous alloys with iron contents as high as 82 at%. J. Magn. Magn. Mater..

[18] Li W., Yang Y.Z., Xu J. (2017). Crystallization and soft magnetic properties of metalloid-free Fe_89_Hf_7_Al_3_Zr_1_ amorphous alloy. J. Non Cryst. Solids.

[19] Trexler M.M., Thadhani N.N. (2010). Mechanical properties of bulk metallic glasses. Prog. Mater. Sci..

[20] Ishida M., Takeda H., Watanabe D., Amiya K., Nishiyama N., Kita K., Saotome Y., Inoue A. (2004). Fillability and Imprintability of High-strength Ni-based Bulk Metallic Glass Prepared by the Precision Die-casting Technique. Mater. Trans..

[21] Narayan R.L., Singh P.S., Hofmann D.C., Hutchinson N., Flores K.M., Ramamurty U. (2012). On the microstructure–tensile property correlations in bulk metallic glass matrix composites with crystalline dendrites. Acta Mater..

[22] Si J., Mei J., Wang R., Chen X., Hui X. (2016). Fe-B-Si-Zr bulk metallic glasses with ultra high compressive strength and excellent soft magnetic properties. Mater. Lett..

[23] Pietrusiewicz P. (2018). Magnetic Properties of the Rapidly Solidified Bulk Alloy: Fe_61_Co_10_B_20_Y_8x_W_y_Pt_x_ (where: x = 1; 2; y = 0; 1). Acta Phys. Pol. A.

[24] Liu T., Li F., Wang A., Xie L., He Q., Luan J., He A., Wang X., Liu C.T., Yang Y. (2019). High performance Fe-based nanocrystalline alloys with excellent thermal stability. J. Alloy Compd..

[25] Jung H.Y., Yi S. (2014). Nanocrystallization and soft magnetic properties of Fe_23_M_6_ (M:C or B) phase in Fe-based bulk metallic glass. Intermetallics.

[26] Elmanov G.N., Chernavskii P.A., Kozlov I.V., Dzhumaev P.S., Kostitsyna E.V., Tarasov V.P., Ignatov A.S., Gudoshnikov S.A. (2018). Effect of heat treatment on phase transformations and magnetization of amorphous Co_69_Fe_4_Cr_4_Si_12_B_11_ microwires. J. Alloy Compd..

[27] Xia G.T., Wang Y.G., Dai J., Dai Y.D. (2017). Effects of Cu cluster evolution on soft magnetic properties of Fe_83_B_10_C_6_Cu_1_ metallic glass in two-step annealing. J. Alloy Compd..

[28] Wang W.H. (2007). Roles of minor additions in formation and properties of bulk metallic glasses. Prog. Mater. Sci..

[29] Nabiałek M. (2015). Soft magnetic and microstructural investigation in Fe-based amorphous alloy. J. Alloy Compd..

[30] Chen H.S., Turnbull D. (1969). Formation, stability and structure of palladium-silicon based alloy glasses. Acta Metall..

[31] Pietrusiewicz P., Nabiałek M. (2017). Analysis of the Thermal and Magnetic Properties of Amorphous Ribbons of Fe_61_Co_10_B_20_Y_8_Me_1_ (where Me = W, Zr, Nb, Mo). Acta Phys. Pol. A.

[32] Oesterreicher H., Abache C. (1985). Fe//1//4R//2B type compounds for magnetic recording applications. J. Phys. Colloq..

[33] Zhai X.B., Wang Y.G., Zhu L., Cao C.C., Dai Y.D., Chen J.K., Pan F.M. (2018). Effect of heating rate on atom migration, phase structure and magnetic properties of the Fe_82_Si_2_B_11_P_4_Cu_1_ alloy. J. Non Cryst. Solids.

[34] Gondro J., Błoch K., Nabiałek M., Walters K., Szota M. (2015). Microstructure And Magnetic Properties Of The FeZr (Y) NbCuB Amorphous Alloys. Arch. Metall. Mater..

[35] Nabiałek M., Jeż B., Błoch K., Pietrusiewicz P., Gondro J. (2019). The effect of the cobalt-content on the magnetic properties of iron-based amorphous alloys. J. Magn. Magn. Mater..

[36] Nabiałek M., Jeż K. (2019). Approach to the ferromagnetic saturation of the Fe_39_Co_33_Y_8_B_20_ alloy produced using two methods. Eur. J. Mater. Sci. Eng..

[37] Imafuku M., Sato S., Koshiba H., Matsubara E., Inoue A. (2001). Structural study of Fe-Based glassy alloys with a large super cooled liquid region. Mater. Res. Soc. Symp. Proc..

[38] Martinez-Garcia J.C., Garcia J.C., Rivas M. (2008). Asymmetric magnetization reversal of partially devitrified Co_66_Si_15_B_14_Fe_4_Ni_1_. J. Non Cryst. Solids.

[39] Bennet L.H., Torre E.D. (2005). Analysis of wasp-waist hysteresis loops. J. Appl. Phys..

[40] Kulik T. (1998). Soft Magnetic Nanocrystalline Materials Obtained by Crystallization of Metallic Glasses.

[41] Gruszka K. (2013). Properties of Bulk Nanocrystalline Materials Obtained in One-Stage Manufacturing Process and after Isothermal Heating. Ph.D. Thesis.

[42] Nabiałek M., Dospiał M., Szota M. (2010). Study of the mechanical and magnetic properties of Fe_61_Co_10_Zr_5-x_Hf_x_W_2_Y_2_B_20_ (x = 0 or 3) bulk amorphous and crystalline alloys. Phys. Status Solidi C.

